# Crystal structure of 4-[(3-meth­oxy-2-oxidobenzyl­idene)azaniumyl]­benzoic acid methanol monosolvate

**DOI:** 10.1107/S2056989018016262

**Published:** 2018-11-22

**Authors:** Saima Kamaal, Md. Serajul Haque Faizi, Akram Ali, Musheer Ahmad, Turganbay Iskenderov

**Affiliations:** a Department of Applied Chemistry, Faculty of Engineering & Technology, Aligarh, Muslim University, Aligarh UP 202002, India; b Department of Chemistry, Langat Singh College, B. R. A. Bihar University, Muzaffarpur, Bihar 842 001, India; c CMP College Allahabad, a constitution college of Allahabad University, Allahabad, UP, India; d National Taras Shevchenko University, Department of Chemistry, Volodymyrska str., 64, 01601 Kyiv, Ukraine

**Keywords:** crystal structure, zwitterion, 2-hy­droxy-3-meth­oxy-benzaldehyde, 4-amino­benzoic acid (PABA), Schiff base, hydrogen bonding, vanillin

## Abstract

In the crystal of the title compound, C_15_H_13_NO_4_·CH_3_OH, the Schiff base mol­ecule exists in the zwitterionic form; an intra­molecular N—H⋯O hydrogen bond stabilizes the mol­ecular structure. The benzene rings are nearly co-planar, subtending a dihedral angle of 5.34 (2)°. In the crystal, classical O—H⋯O and weak C—H⋯O hydrogen bonds link the Schiff base mol­ecules and methanol solvent mol­ecules into a three-dimensional supra­molecular architecture. The crystal studied was refined as an inversion twin.

## Abstract 2

In the crystal, the Schiff base mol­ecule exists in the zwitterionic form and an intra­molecular N—H⋯O hydrogen bond stabilizes the mol­ecular structure.

## Chemical context   

Vanillin and *o*-vanillin are natural compounds that have both a phenolic OH and an aldehyde group. They are positional isomers, in which *o*-vanillin shows contradictory effects. There are several reports indicating that *o*-vanillin induces mutations and it has also been found to enhance chromosomal aberrations in *in vitro* systems (Barik *et al.*, 2004 ▸; Takahashi *et al.*, 1989 ▸). Vanillin is also the primary component of the extract of the vanilla bean. Synthetic vanillin rather than natural vanilla extract is now more often used as a flavouring agent in foods, beverages and pharmaceuticals. Schiff bases containing *o*-vanillin possess anti­fungal and anti­bacterial properties (Thorat *et al.*, 2012 ▸). 4-Amino­benzoic acid (PABA) is an important biological mol­ecule, being an essential bacterial cofactor involved in the synthesis of folic acid (Robinson, 1966 ▸). PABA shows polymorphism and so far four polymorphs of PABA are known, all of which are centrosymmetric; a non-centrosymmetric polymorph of 4-amino­benzoic acid has also been reported (Benali-Cherif *et al.*, 2014 ▸). Schiff bases derived from 2-hy­droxy-3-meth­oxy­benzaldehyde (*o*-vanillin) and PABA have not been investigated so thoroughly. Our research inter­est focuses on the study of Schiff bases derived from salicyl­aldehyde. It is well known that Schiff bases of salicyl­aldehyde derivatives may exhibit thermochromism or photochromism, depending on the planarity or non-planarity of the mol­ecule (Cohen & Schmidt, 1964 ▸; Amimoto & Kawato, 2005 ▸). Schiff bases often exhibit various biological activities and in many cases have been shown to possess anti­bacterial, anti­cancer, anti-inflammatory and anti­toxic properties (Lozier *et al.*, 1975 ▸). They are used as anion sensors (Dalapati *et al.*, 2011 ▸), as non-linear optical compounds (Sun *et al.*, 2012 ▸) and as versatile polynuclear ligands for multinuclear magnetic exchange clusters (Moroz *et al.*, 2012 ▸). New salicyl­aldehyde-based Schiff bases have also been synthesized and reported (Faizi *et al.*, 2015*a* ▸,*b* ▸; 2016*b* ▸; 2017*a* ▸,*b* ▸,*c* ▸). The present work is a part of an ongoing structural study of Schiff bases and their utilization in the synthesis of new organic, excited state proton-transfer compounds and fluorescent chemosensors (Faizi *et al.*, 2016*a* ▸; Faizi *et al.*, 2018 ▸; Kumar *et al.*, 2018 ▸; Mukherjee *et al.*, 2018 ▸). We report herein the crystal structure of the title compound synthesized by the condensation reaction of 2-hy­droxy-3-meth­oxy­benzaldehyde and PABA.
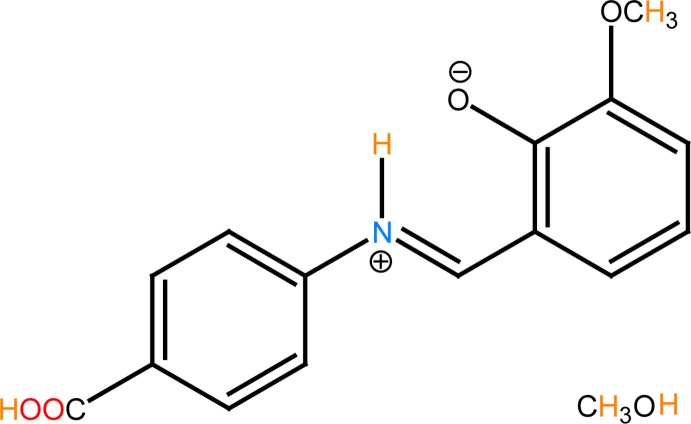


## Structural commentary   

**Figure 1:**
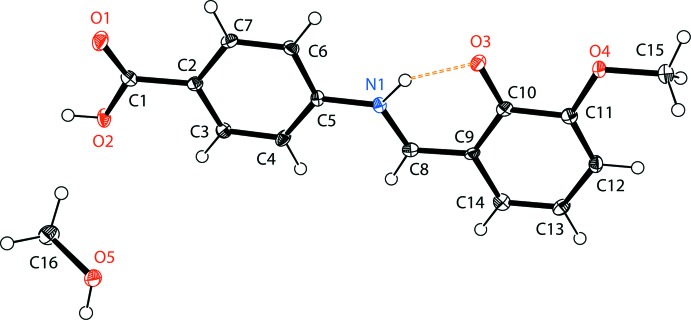
The mol­ecular structure of the title compound, showing the atom labelling and the intra­molecular N—H⋯O hydrogen bond as a dashed line. Displacement ellipsoids are drawn at the 40% probability level.

The asymmetric unit of the title compound contains a Schiff base mol­ecule and a methanol mol­ecule of crystallization. In the solid state, the Schiff base mol­ecule (Fig. 1 ▸) exists in the zwitterionic form. An intra­molecular N—H⋯O hydrogen bond stabilizes the mol­ecular structure (Table 1 ▸). The imine group, which displays a C9—C8—N1—C5 torsion angle of 177.6 (3)°, contributes to the general planarity of the mol­ecule. The Schiff base mol­ecule displays a *trans* configuration with respect to the C=N and C–N bonds. The vanillin ring (C9–C14) is inclined to the central benzene ring (C2–C7) by 5.34 (2)°. A similar value of 5.3 (2)° is observed in 4-chloro-*N*′-(2-hy­droxy-4-meth­oxy­benzyl­idene)benzohydrazide meth­anol monosolvate (Zhi *et al.*, 2011 ▸). All bond lengths are in normal ranges. The O4—C15 bond length is 1.432 (2) Å and similar value of 1.432 (2) Å is observed in (*E*)-2-hy­droxy-3-meth­oxy-5-[(3-meth­oxy­phen­yl)diazen­yl]benzaldehyde (Karadayı *et al.*, 2006 ▸). The meth­oxy group of the 2-hy­droxy-3-meth­oxy­phenyl is almost coplanar with its bound benzene ring, as seen by the C_meth­yl_—O—C—C torsion angle of 178.1 (2)°.

## Supra­molecular features   

**Figure 2:**
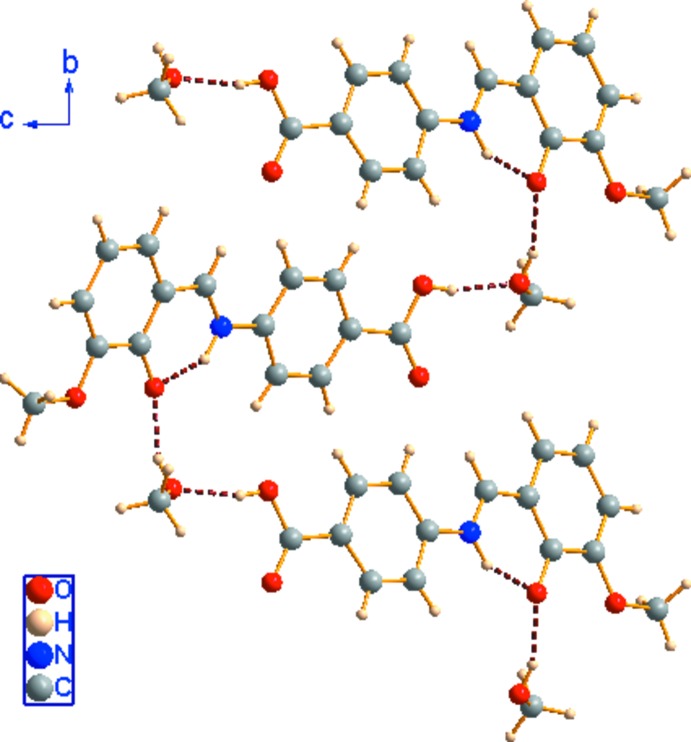
A view of the hydrogen-bonded chain extending along the *b-*axis direction. Hydrogen bonds are shown as dashed lines.

In the crystal, the hydroxyl group of the methanol solvent mol­ecule is linked to the carboxyl­ate group of the neighboring Schiff base mol­ecule and the deprotonated hydroxyl group of the other Schiff base mol­ecule via classical O—H⋯O hydrogen bonds, forming supra­molecular chains propagating along the *b*-axis direction (Fig. 2 ▸). Weak C—H⋯O hydrogen bonds further link the chains into a three-dimensional supra­molecular architecture.

## Database survey   

**Figure 3:**
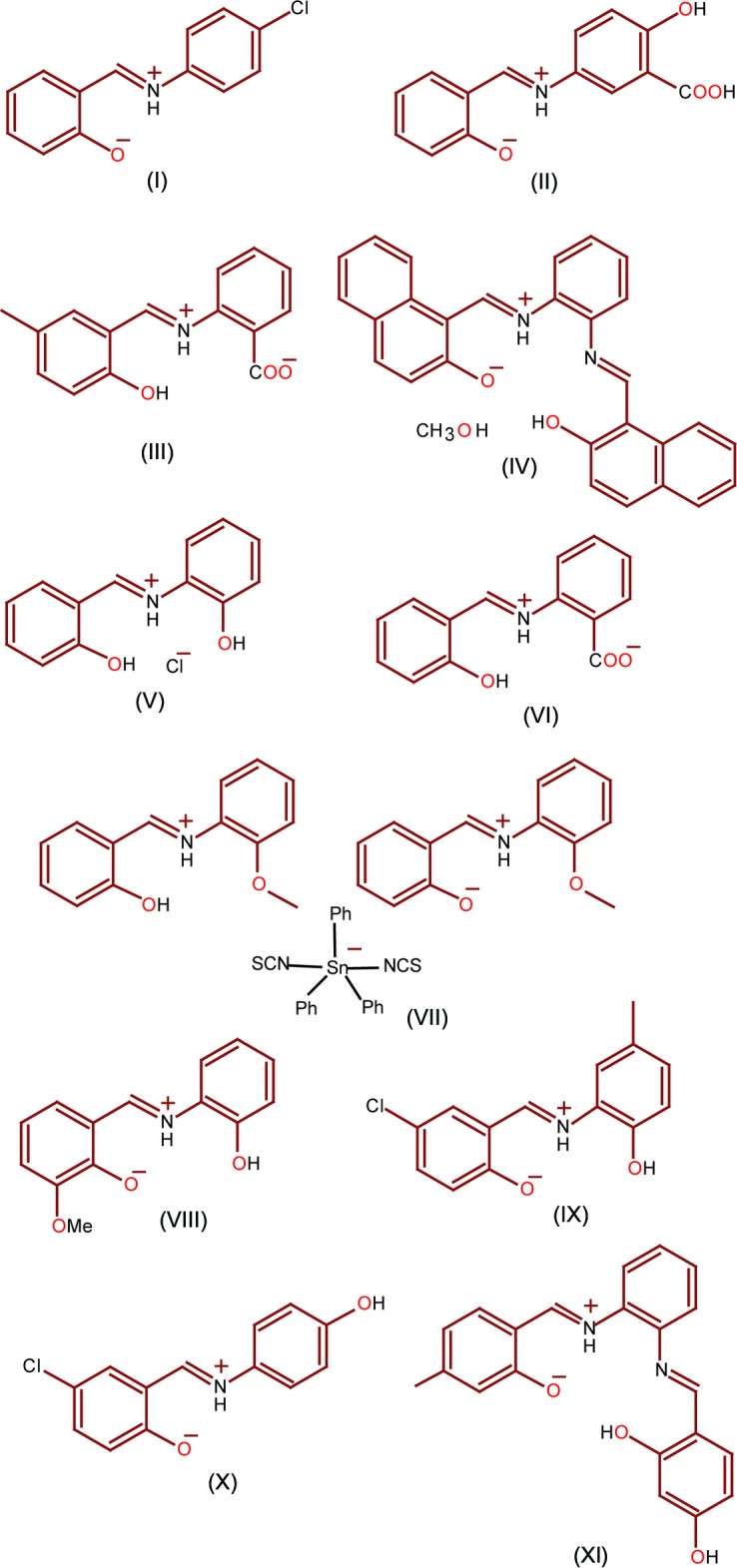
Zwitterionic forms of some closely related compounds.

A search of the Cambridge Structural Database (CSD version 5.39, February 2018 update; Groom *et al.*, 2016 ▸) for similar systems (benzyl­idene-phenyl-amine) yielded 285 hits of which ten are similar substituted benzyl­idene-phenyl-amines: *N*-salicyl­idene-*p*-chloro­aniline (I) (BADDAL01; Kamwaya & Khoo, 1985 ▸), 5-{[(1*E*)-(2-hy­droxy­phen­yl)methyl­ene]amino}-2-hy­droxy­benzoic acid (II) (CAWJOA; Bourque *et al.*, 2005 ▸), 2-(2-hy­droxy-5-methyl­benzyl­idene­ammonio)­benzoate (III) (CEXNEZ; Gayathri *et al.*, 2007 ▸), *N*,*N*′-bis­(2-hy­droxy-1-naphthaldimine)-*o*-phenyl­enedi­amine methanol solvate (IV) (GETXEJ; Eltayeb *et al.*, 2007 ▸), *o*-(salicylideneaminium)phenol chloride (V) (HALGUW; Ondrácek *et al.*, 1993 ▸), *N*-(2-carb­oxy­phen­yl)salicylidenimine (VI) (JUTKAK; Ligtenbarg *et al.*, 1999 ▸), diiso­thio­cyantotri­phenyl­tin bis­[1-(salicycl­idene­imino)-2-meth­oxy­benzene] (VII) (KIDYOL; Charland *et al.*, 1989 ▸), *N*-(2-oxyphen­yl)-3-meth­oxy­salicylaldimine (VIII) (NEDMUF; Kannappan *et al.*, 2006 ▸), *N*-(5-chloro-2-oxido­benzyl­idene)-2-hy­droxy-5-methyl­anilinium (IX) (QIKHEX; Elmali *et al.*, 2001 ▸) and *N*-(5-chloro-2-hy­droxy­benzyl­idene)-4-hy­droxy­aniline (X) (SAQTOT; Ogawa *et al.*, 1998 ▸), 2-[(*E*)-(2-[{(*E*)-2,3-di­hydroxy­benzyl­idene]amino}-5-methyl­phen­yl)iminiometh­yl]-6-hy­droxy­phenolate (XI) (HUCQEC; Eltayeb *et al.*, 2009 ▸) (see Fig. 3 ▸). The dihedral angle between the benzene rings in the title compound [5.34 (2)°] is smaller than those in compounds (III) [5.6 (1)°] (IV [5.84 (9)°], (V) [7.3 (1)°] and (IX) [9.51 (6)°] and (XI) [17.36 (12)°]. In compound (VII), cationic protonated pairs co-crystallize with five-coordinate organotin anions. In the title compound, they form an intra­molecular *S*6 ring motif and stabilized by N—H⋯O hydrogen bonds.

## Synthesis and crystallization   

To a hot stirred solution of 4-amino­benzoic acid (PABA) (1.00 g, 7.2 mmol) in methanol (15 ml) was added vanillin (1.11 g, 7.2 mmol)). The resulting mixture was then heated under reflux. After an hour, a precipitate formed. The reaction mixture was heated for about another 30 min until the completion of the reaction, which was monitored by TLC. The reaction mixture was cooled to room temperature, filtered and washed with hot methanol. It was then dried under vacuum to give the pure compound in 78% yield. Prismatic colourless single crystals of the title compound suitable for X-ray analysis were obtained by slow evaporation of a methanol solution.

## Refinement   

Crystal data, data collection and structure refinement details are summarized in Table 2 ▸. The N—H and O–H atoms were located in a difference-Fourier map. Their positional and isotropic thermal parameters were included in further stages of the refinement. All C-bound H atoms were positioned geometrically and refined using a riding model with C—H = 0.93–0.97 Å and with *U*_iso_(H) = 1.2–1.5*U*_eq_(C).

## Supplementary Material

Crystal structure: contains datablock(s) I, global. DOI: 10.1107/S2056989018016262/xu5950sup1.cif

Structure factors: contains datablock(s) I. DOI: 10.1107/S2056989018016262/xu5950Isup2.hkl

CCDC reference: 1879300

Additional supporting information:  crystallographic information; 3D view; checkCIF report

## Figures and Tables

**Table 1:** Hydrogen-bond geometry (Å, °)

*D*—H⋯*A*	*D*—H	H⋯*A*	*D*⋯*A*	*D*—H⋯*A*
N1—H1⋯O3	0.86	1.87	2.568 (4)	138
O2—H2⋯O5^i^	0.82	1.80	2.598 (4)	164
O5—H5*O*⋯O3^ii^	0.96 (5)	1.77 (5)	2.690 (4)	159 (4)
C7—H7⋯O2^i^	0.93	2.56	3.233 (5)	130
C8—H8⋯O1^iii^	0.93	2.41	3.281 (5)	155

Symmetry codes: (i) 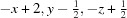; (ii) 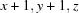; (iii) 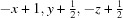.

**Table 2:** Experimental details

Crystal data	
Chemical formula	C_15_H_13_NO_4_·CH_4_O
*M*_r_	303.30
Crystal system, space group	Orthorhombic, *P*2_1_2_1_2_1_
Temperature (K)	296
*a*, *b*, *c* (Å)	4.6993 (5), 10.038 (1), 30.155 (3)
*V* (Å^3^)	1422.5 (3)
*Z*	4
Radiation type	Mo *K*α
μ (mm^−1^)	0.11
Crystal size (mm)	0.61 × 0.36 × 0.17

Data collection	
Diffractometer	Stoe IPDS 2
Absorption correction	Integration (*X-RED32*; Stoe & Cie, 2002 ▸)
*T*_min_, *T*_max_	0.963, 0.988
No. of measured, independent and observed [*I* > 2σ(*I*)] reflections	17046, 2526, 2117
*R*_int_	0.095
(sin θ/λ)_max_ (Å^−1^)	0.596

Refinement	
*R*[*F* ^2^ > 2σ(*F* ^2^)], *wR*(*F* ^2^), *S*	0.046, 0.112, 1.08
No. of reflections	2526
No. of parameters	206
H-atom treatment	H atoms treated by a mixture of independent and constrained refinement
Δρ_max_, Δρ_min_ (e Å^−3^)	0.25, −0.26
Absolute structure	Refined as a perfect inversion twin.
Absolute structure parameter	0.5

Computer programs: *X-AREA* and *X-RED* (Stoe & Cie, 2002 ▸), *SHELXT2014* (Sheldrick, 2015*a* ▸), *SHELXL2017* (Sheldrick, 2015*b* ▸), *ORTEP-3 for Windows* and *WinGX* (Farrugia, 2012 ▸) and *PLATON* (Spek, 2009 ▸).

## supplementary crystallographic information

### Crystal data

**Table:**

C_15_H_13_NO_4_·CH_4_O	*D*_x_ = 1.416 Mg m^−^^3^
*M**_r_* = 303.30	Mo *K*α radiation, λ = 0.71073 Å
Orthorhombic, *P*2_1_2_1_2_1_	Cell parameters from 8708 reflections
*a* = 4.6993 (5) Å	θ = 2.4–29.9°
*b* = 10.038 (1) Å	µ = 0.11 mm^−^^1^
*c* = 30.155 (3) Å	*T* = 296 K
*V* = 1422.5 (3) Å^3^	Prism, colorless
*Z* = 4	0.61 × 0.36 × 0.17 mm
*F*(000) = 640	

### Data collection

**Table:**

STOE IPDS 2 diffractometer	2526 independent reflections
Radiation source: sealed X-ray tube, 12 x 0.4 mm long-fine focus	2117 reflections with *I* > 2σ(*I*)
Plane graphite monochromator	*R*_int_ = 0.095
Detector resolution: 6.67 pixels mm^-1^	θ_max_ = 25.1°, θ_min_ = 2.7°
rotation method scans	*h* = −5→5
Absorption correction: integration (X-RED32; Stoe & Cie, 2002)	*k* = −11→11
*T*_min_ = 0.963, *T*_max_ = 0.988	*l* = −35→35
17046 measured reflections	

### Refinement

**Table:**

Refinement on *F*^2^	Hydrogen site location: mixed
Least-squares matrix: full	H atoms treated by a mixture of independent and constrained refinement
*R*[*F*^2^ > 2σ(*F*^2^)] = 0.046	*w* = 1/[σ^2^(*F*_o_^2^) + (0.0401*P*)^2^ + 0.7153*P*] where *P* = (*F*_o_^2^ + 2*F*_c_^2^)/3
*wR*(*F*^2^) = 0.112	(Δ/σ)_max_ < 0.001
*S* = 1.08	Δρ_max_ = 0.25 e Å^−^^3^
2526 reflections	Δρ_min_ = −0.26 e Å^−^^3^
206 parameters	Absolute structure: Refined as a perfect inversion twin.
0 restraints	Absolute structure parameter: 0.5

### Special details

**Table:**

Geometry. All esds (except the esd in the dihedral angle between two l.s. planes) are estimated using the full covariance matrix. The cell esds are taken into account individually in the estimation of esds in distances, angles and torsion angles; correlations between esds in cell parameters are only used when they are defined by crystal symmetry. An approximate (isotropic) treatment of cell esds is used for estimating esds involving l.s. planes.
Refinement. Refined as a two-component inversion twin

### Fractional atomic coordinates and isotropic or equivalent isotropic displacement parameters (Å^2^)

**Table:**

	*x*	*y*	*z*	*U*_iso_*/*U*_eq_	
C1	0.9825 (8)	0.5743 (4)	0.20579 (12)	0.0150 (8)	
C2	0.8025 (8)	0.5849 (4)	0.24609 (12)	0.0135 (8)	
C3	0.6780 (8)	0.7039 (4)	0.25889 (12)	0.0156 (9)	
H3	0.723443	0.781513	0.243631	0.019*	
C4	0.4888 (8)	0.7099 (4)	0.29364 (12)	0.0168 (9)	
H4	0.407880	0.790869	0.301780	0.020*	
C5	0.4194 (8)	0.5940 (4)	0.31650 (11)	0.0117 (8)	
C6	0.5553 (8)	0.4750 (4)	0.30576 (12)	0.0161 (8)	
H6	0.519516	0.398578	0.322296	0.019*	
C7	0.7427 (8)	0.4703 (4)	0.27069 (12)	0.0163 (9)	
H7	0.830172	0.390229	0.263318	0.020*	
C8	0.0577 (8)	0.6914 (4)	0.36404 (12)	0.0137 (8)	
H8	0.082942	0.774555	0.351007	0.016*	
C9	−0.1484 (8)	0.6767 (4)	0.39755 (12)	0.0133 (8)	
C10	−0.1987 (8)	0.5486 (4)	0.41710 (12)	0.0134 (8)	
C11	−0.4107 (8)	0.5431 (4)	0.45140 (12)	0.0149 (9)	
C12	−0.5604 (9)	0.6534 (4)	0.46335 (12)	0.0162 (8)	
H12	−0.697197	0.646746	0.485520	0.019*	
C13	−0.5121 (8)	0.7778 (4)	0.44273 (12)	0.0175 (9)	
H13	−0.618318	0.851778	0.451168	0.021*	
C14	−0.3108 (8)	0.7897 (4)	0.41061 (12)	0.0162 (9)	
H14	−0.279196	0.871695	0.397153	0.019*	
C15	−0.6548 (9)	0.4036 (4)	0.50242 (13)	0.0216 (10)	
H15A	−0.838233	0.422645	0.489935	0.032*	
H15B	−0.617086	0.464455	0.526284	0.032*	
H15C	−0.652337	0.313983	0.513495	0.032*	
C16	0.4392 (9)	1.1681 (4)	0.40083 (13)	0.0226 (9)	
H16A	0.377953	1.093074	0.383559	0.034*	
H16B	0.323804	1.244117	0.393731	0.034*	
H16C	0.420226	1.147815	0.431800	0.034*	
N1	0.2152 (6)	0.5912 (3)	0.35065 (10)	0.0128 (7)	
H1	0.191331	0.516474	0.364068	0.015*	
O1	1.0635 (6)	0.4688 (3)	0.19035 (9)	0.0244 (7)	
O2	1.0413 (6)	0.6916 (2)	0.18743 (8)	0.0185 (6)	
H2	1.128598	0.680031	0.164203	0.028*	
O3	−0.0617 (6)	0.4431 (2)	0.40503 (8)	0.0159 (6)	
O4	−0.4410 (6)	0.4183 (3)	0.46896 (8)	0.0185 (6)	
O5	0.7301 (6)	1.1970 (3)	0.39101 (9)	0.0192 (6)	
H5O	0.795 (11)	1.280 (5)	0.4034 (16)	0.049 (15)*	

### Atomic displacement parameters (Å^2^)

**Table:**

	*U*^11^	*U*^22^	*U*^33^	*U*^12^	*U*^13^	*U*^23^
C1	0.014 (2)	0.016 (2)	0.0152 (19)	−0.0037 (18)	−0.0028 (17)	0.0022 (16)
C2	0.0111 (19)	0.014 (2)	0.0151 (19)	0.0001 (18)	−0.0019 (16)	−0.0005 (16)
C3	0.016 (2)	0.012 (2)	0.0190 (19)	−0.0024 (18)	0.0022 (17)	0.0026 (16)
C4	0.016 (2)	0.0114 (19)	0.022 (2)	0.0007 (18)	0.0053 (18)	−0.0034 (16)
C5	0.0084 (17)	0.016 (2)	0.0110 (18)	−0.0041 (17)	−0.0013 (16)	−0.0014 (15)
C6	0.0147 (19)	0.015 (2)	0.018 (2)	0.0004 (17)	0.0007 (18)	0.0051 (16)
C7	0.017 (2)	0.014 (2)	0.018 (2)	0.0037 (18)	−0.0006 (18)	−0.0011 (16)
C8	0.0125 (18)	0.0132 (19)	0.0155 (19)	−0.0008 (18)	−0.0037 (16)	−0.0013 (15)
C9	0.0106 (18)	0.015 (2)	0.0145 (19)	0.0034 (16)	−0.0026 (16)	−0.0007 (16)
C10	0.0091 (18)	0.018 (2)	0.0132 (18)	−0.0011 (16)	−0.0057 (16)	−0.0018 (16)
C11	0.012 (2)	0.018 (2)	0.0147 (19)	−0.0022 (17)	−0.0030 (16)	−0.0003 (16)
C12	0.0139 (19)	0.022 (2)	0.0125 (19)	−0.0008 (18)	0.0011 (17)	−0.0004 (16)
C13	0.013 (2)	0.019 (2)	0.021 (2)	0.0011 (17)	−0.0017 (18)	−0.0046 (16)
C14	0.015 (2)	0.016 (2)	0.0175 (19)	−0.0052 (17)	−0.0032 (17)	−0.0011 (17)
C15	0.019 (2)	0.024 (2)	0.021 (2)	0.000 (2)	0.0052 (18)	0.0036 (18)
C16	0.016 (2)	0.026 (2)	0.025 (2)	0.0012 (19)	−0.0024 (19)	0.0013 (18)
N1	0.0126 (16)	0.0129 (17)	0.0130 (16)	−0.0029 (15)	−0.0009 (14)	0.0015 (13)
O1	0.0321 (17)	0.0155 (15)	0.0256 (15)	0.0024 (13)	0.0129 (14)	−0.0001 (12)
O2	0.0237 (15)	0.0145 (14)	0.0174 (14)	−0.0034 (13)	0.0081 (13)	0.0000 (11)
O3	0.0147 (13)	0.0149 (14)	0.0182 (13)	0.0010 (12)	0.0029 (12)	−0.0012 (11)
O4	0.0172 (13)	0.0185 (14)	0.0197 (14)	0.0012 (13)	0.0071 (12)	0.0053 (12)
O5	0.0163 (14)	0.0205 (15)	0.0209 (14)	−0.0015 (14)	0.0047 (12)	−0.0042 (13)

### Geometric parameters (Å, º)

**Table:**

C1—O1	1.217 (4)	C8—C9	1.408 (5)
C1—O2	1.331 (4)	C9—C14	1.422 (5)
C1—C2	1.484 (5)	C9—C10	1.434 (5)
C2—C3	1.384 (5)	C10—O3	1.292 (4)
C2—C7	1.397 (5)	C10—C11	1.437 (5)
C3—C4	1.376 (5)	C11—C12	1.360 (5)
C4—C5	1.392 (5)	C11—O4	1.367 (4)
C5—C6	1.393 (5)	C12—C13	1.414 (5)
C5—N1	1.408 (4)	C13—C14	1.359 (5)
C6—C7	1.377 (5)	C15—O4	1.432 (4)
C8—N1	1.312 (5)	C16—O5	1.429 (5)
			
O1—C1—O2	123.1 (3)	C8—C9—C14	119.0 (3)
O1—C1—C2	123.6 (3)	C8—C9—C10	120.2 (3)
O2—C1—C2	113.3 (3)	C14—C9—C10	120.8 (3)
C3—C2—C7	118.5 (3)	O3—C10—C9	122.5 (3)
C3—C2—C1	122.1 (3)	O3—C10—C11	121.1 (3)
C7—C2—C1	119.4 (3)	C9—C10—C11	116.4 (3)
C4—C3—C2	121.6 (4)	C12—C11—O4	126.1 (3)
C3—C4—C5	119.4 (3)	C12—C11—C10	121.2 (3)
C4—C5—C6	119.7 (3)	O4—C11—C10	112.7 (3)
C4—C5—N1	122.6 (3)	C11—C12—C13	121.3 (4)
C6—C5—N1	117.8 (3)	C14—C13—C12	120.1 (4)
C7—C6—C5	120.1 (3)	C13—C14—C9	120.1 (4)
C6—C7—C2	120.6 (3)	C8—N1—C5	126.5 (3)
N1—C8—C9	121.9 (3)	C11—O4—C15	116.1 (3)
			
O1—C1—C2—C3	−169.8 (4)	C8—C9—C10—C11	179.3 (3)
O2—C1—C2—C3	8.6 (5)	C14—C9—C10—C11	−2.6 (5)
O1—C1—C2—C7	6.5 (5)	O3—C10—C11—C12	−178.3 (3)
O2—C1—C2—C7	−175.1 (3)	C9—C10—C11—C12	2.0 (5)
C7—C2—C3—C4	−3.0 (6)	O3—C10—C11—O4	1.1 (5)
C1—C2—C3—C4	173.3 (3)	C9—C10—C11—O4	−178.6 (3)
C2—C3—C4—C5	−0.2 (6)	O4—C11—C12—C13	−179.7 (3)
C3—C4—C5—C6	4.0 (6)	C10—C11—C12—C13	−0.4 (6)
C3—C4—C5—N1	−176.2 (3)	C11—C12—C13—C14	−0.7 (6)
C4—C5—C6—C7	−4.5 (6)	C12—C13—C14—C9	0.2 (5)
N1—C5—C6—C7	175.7 (3)	C8—C9—C14—C13	179.7 (3)
C5—C6—C7—C2	1.2 (6)	C10—C9—C14—C13	1.6 (5)
C3—C2—C7—C6	2.5 (6)	C9—C8—N1—C5	177.6 (3)
C1—C2—C7—C6	−173.9 (3)	C4—C5—N1—C8	3.2 (6)
N1—C8—C9—C14	179.7 (3)	C6—C5—N1—C8	−176.9 (3)
N1—C8—C9—C10	−2.2 (5)	C12—C11—O4—C15	1.2 (5)
C8—C9—C10—O3	−0.4 (5)	C10—C11—O4—C15	−178.1 (3)
C14—C9—C10—O3	177.7 (3)		

### Hydrogen-bond geometry (Å, º)

**Table:**

*D*—H···*A*	*D*—H	H···*A*	*D*···*A*	*D*—H···*A*
N1—H1···O3	0.86	1.87	2.568 (4)	138
O2—H2···O5^i^	0.82	1.80	2.598 (4)	164
O5—H5*O*···O3^ii^	0.96 (5)	1.77 (5)	2.690 (4)	159 (4)
C7—H7···O2^i^	0.93	2.56	3.233 (5)	130
C8—H8···O1^iii^	0.93	2.41	3.281 (5)	155

Symmetry codes: (i) −*x*+2, *y*−1/2, −*z*+1/2; (ii) *x*+1, *y*+1, *z*; (iii) −*x*+1, *y*+1/2, −*z*+1/2.
